# Scleritis and anterior uveitis may herald the development of an epibulbar tumor in patients with extranodal Rosai-Dorfman disease: a case report

**DOI:** 10.1186/s12886-019-1158-2

**Published:** 2019-07-10

**Authors:** Yu-Kuei Lee, Sheau-Chiou Chao, Chaw-Ning Lee, Jia-Horung Hung

**Affiliations:** 1 Department of Ophthalmology, National Cheng Kung University Hospital, College of Medicine, National Cheng Kung University, Tainan, Taiwan; 2 Department of Dermatology, National Cheng Kung University Hospital, College of Medicine, National Cheng Kung University, Tainan, Taiwan; 3 Institute of Clinical Medicine, College of Medicine, National Cheng Kung University, Tainan, Taiwan

**Keywords:** Rosai-Dorfman disease, Epibulbar tumor, Histiocytosis

## Abstract

**Background:**

Rosai-Dorfman disease is a rare non–Langerhans cell histiocytosis. Ocular involvement is even rarer, mostly involving the orbit and eyelids, although marginal corneal ulcers, uveitis, and epibulbar masses have also been reported, and is characterized by multiple recurrences. However, the disease course and optimal treatment strategies remain undetermined, in light of the rarity of this disease.

**Case presentation:**

We reported a 36-year-old male patient with the extranodal form of Rosai-Dorfman disease, presenting with scleritis and anterior uveitis in the left eye, who experienced subsequent development of an epibulbar tumor in the same eye. The patient was also complicated by a relapsing facial nodule on the right cheek. After the pathological diagnosis of Rosai-Dorfman disease was obtained, the patient underwent surgical excision of the epibulbar tumor and the facial nodule, accompanied by systemic immunosuppression therapy. At the last follow-up, the patient was asymptomatic without signs of recurrence.

**Conclusions:**

This report highlights the progression of ocular manifestations of Rosai-Dorfman disease and emphasizes the importance of systemic therapy.

## Background

Rosai-Dorfman disease (RDD) is a rare non–Langerhans cell histiocytosis that is characterized by the accumulation of activated histiocytes within affected tissues, and it usually presents with prominent cervical lymphadenopathy [1]. Ocular involvement of RDD is even rarer and mostly involves the orbit and eyelids, although marginal corneal ulcers, uveitis, and epibulbar masses have also been reported [1–4]. Herein, we report an unusual case of RDD with ocular and dermatological findings, including scleritis and anterior uveitis and the subsequent development of an epibulbar tumor, and was complicated by a relapsing facial nodule.

## Case presentation

**Fig. 1:**
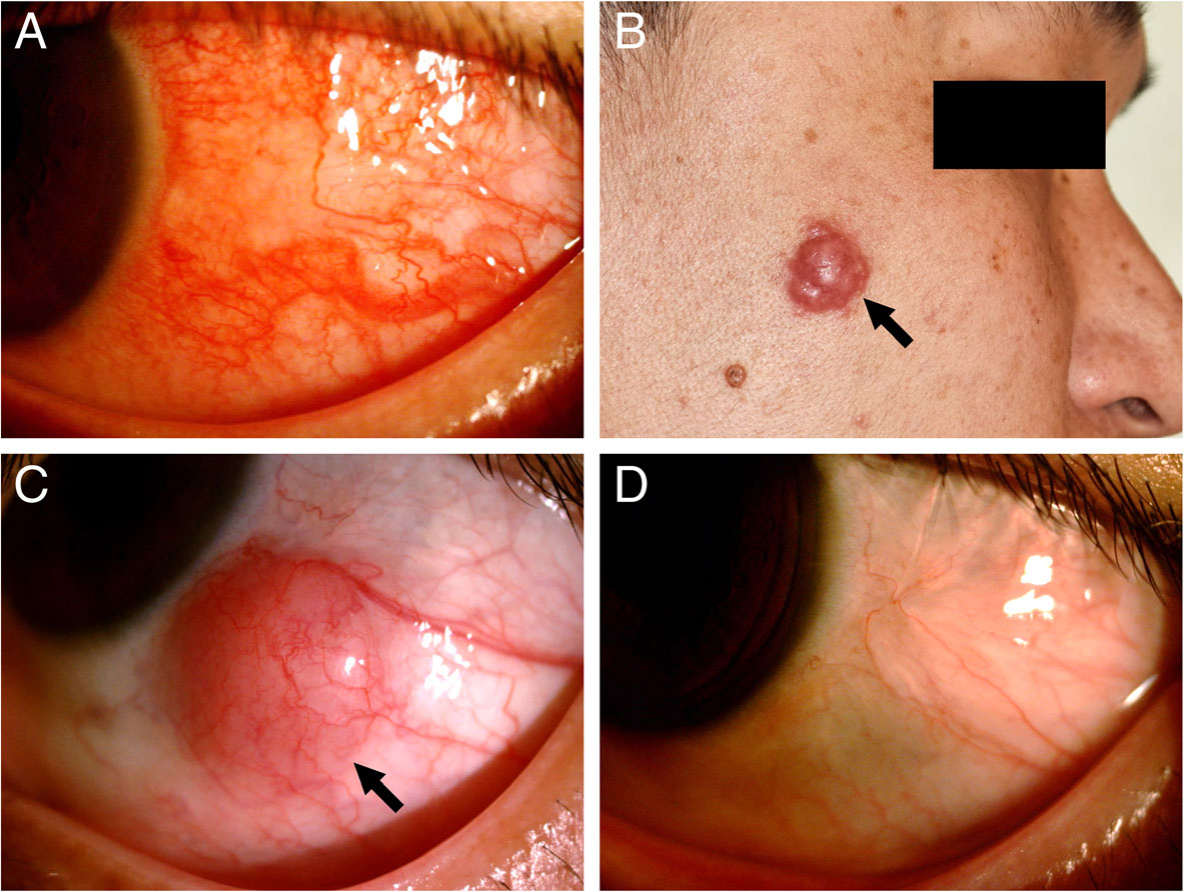
Clinical appearance of Rosai-Dorfman disease (RDD) with ocular and dermatological involvement in a 36-year-old male: (**a**) Slit-lamp photograph of his left eye demonstrating engorged conjunctival vessels and a significantly injected bulbar conjunctiva. **b** RDD of the skin showing a 1.5 × 1.3-cm erythematous soft lobulated nodule, over right face (arrow) on the right cheek. **c** Clinical photographs depicting a well-circumscribed, epibulbar nodule (arrow) at the inferior-temporal aspect of the eyeball after six-months of follow-up. Note that the location of the nodule is essentially similar to that of the scleritis observed in Panel A. **d** Clinical appearance of the left eye showing a quiet conjunctiva without evidence of tumor recurrence at follow-up 24 months later

A 36-year-old male with an unremarkable medical history presented to the ophthalmology service with a 1-month history of a congested and painful left eye accompanied by a persistent left-sided headache. Ophthalmologic examination revealed a best corrected visual acuity of 20/20 for both eyes and an intraocular pressure of 20 and 15 mmHg for the right and left eyes, respectively. Biomicroscopy revealed significant conjunctival injection with engorged vessels in the temporal aspect of the patient’s left eye (Fig. 1a), which did not blanch after instillation of 10% phenylephrine. The cornea was clear, and 3+ cells were visualized in the left anterior chamber. The results of a dilated fundoscopic examination were normal. Laboratory studies demonstrated an elevated C-reactive protein level (25.7 mg/L) and erythrocyte sedimentation rate (32 mm/h). A thorough rheumatologic evaluation was unrevealing, and the following tests were normal, including total and differential white blood cell counts, rheumatoid factor, antinuclear antibodies, antineutrophil cytoplasmic antibodies, serology for syphilis and chest X-ray. A diagnosis of scleritis was made, and the patient received 40 mg oral prednisolone daily and topical 1% prednisolone 4 times daily. Though the ocular symptoms improved, the resolution was incomplete.

Four weeks later, the patient was referred to the dermatology service because physical examination revealed a rapidly-growing erythematous soft lobulated nodule (1.5 × 1.3 cm) on the right side of the patient’s face (Fig. 1b). An incisional biopsy specimen was obtained, and the pathological findings showed dense diffuse nodular infiltrate of epithelioid and multinucleated histiocytes with S-100 positivity mixed with neutrophils throughout the upper two-thirds of the dermis, which was consistent with atypical Rosai-Dorfman disease with abundant neutrophils. The facial nodule continued to grow while the patient was maintained on low-dose prednisolone (5 mg daily). Although intralesional triamcinolone injections were performed weekly for 5 weeks, the tumor continued to enlarge to 4 × 2.5 cm, so excision of the facial skin tumor was performed. The pathological diagnosis was RDD with excessive *Demodex* mites. The maintenance therapy included prednisolone and doxycycline along with *prn* intralesional triamcinolone injections.

**Fig. 2:**
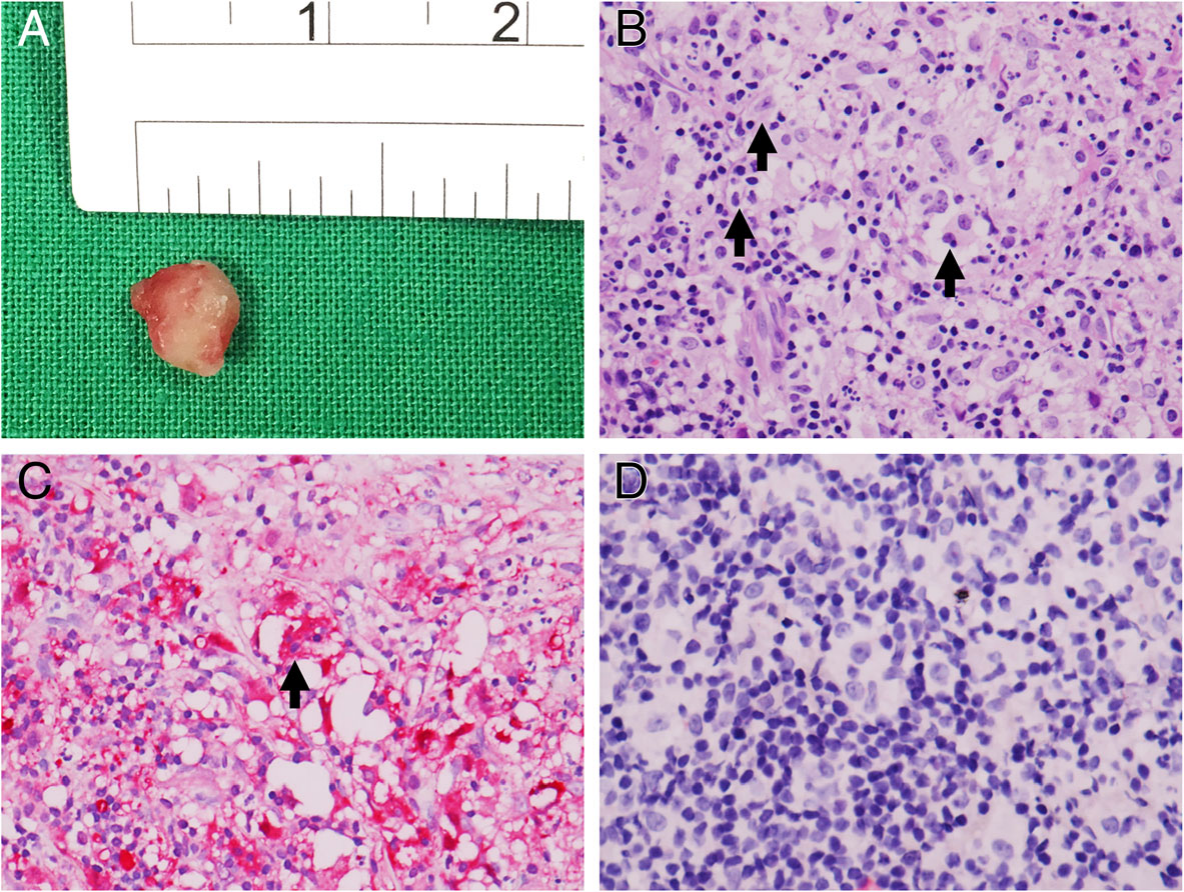
Pathological study of Rosai-Dorfman disease (RDD). **a** The excisional epibulbar nodule (Gross view). **b** Photomicrographs revealing RDD histiocytes with emperipolesis (arrows) (hematoxylin and eosin stain; original magnification, × 400). **c**, **d** Immunohistochemical photomicrograph displaying the histiocytes with positive nuclear and cytoplasmic staining for S-100 (**c**) but negative staining for CD1a (**d**). The arrow in Panel **c** marks emperipolesis (original magnification, × 400)

Two months after the skin surgery, a painless, fixed, pink subconjunctival nodule was noted in the inferior-temporal aspect of his left eye (Fig. 1c), and surgical excision was scheduled. Pathological examination of the specimen demonstrated histiocytes with emperipolesis, cytoplasmic and nuclear S100 positivity, and a negative stain for CD1a, which were also compatible with RDD (Fig. 2a-d). In addition, 1 week after the ocular surgery, multiple discrete and confluent papulonodules rapidly evolved over the bilateral cheek, ear and scalp. Oral dapsone 100 mg daily was administered for 6 weeks and was then switched to methotrexate (MTX) 10 mg once weekly due to poor response. The patient underwent another excision of the tumor on the right side of his face and intermittent intralesional triamcinolone injections. Finally, the lesions gradually flattened, and MTX was then slowly tapered to a maintenance dose of 2.5 mg per week after 4 months. At the 24-month follow-up, there were no signs of recurrence of the epibulbar tumor and facial mass or of involvement of other sites (Fig. 1d).

## Discussion and conclusions

In this case report, we presented the successful management of a case with complex RDD that involved ocular and dermatological aspects. In addition, scleritis and anterior uveitis may foreshadow the development of an epibulbar tumor.

Ophthalmic involvement occurs in approximately 10% of patients with RDD [2, 5]. In a recent review by Choi et al*,* 75% of ocular RDD cases presented with epibulbar masses [2]. Epibulbar masses were first reported as ophthalmic manifestations in RDD by Zimmerman et al in 1988 in a 13-year-old male patient [6]. Since then, several cases have been reported, of which 13% (2 of 15 cases) also had dermatological presentations (Table 1) [2–4, 6–12]. Only one case of RDD has been reported with findings similar to the present case [9]. The ocular RDD presented with scleritis as the initial stage and was characterized by an incomplete response to corticosteroid treatment followed by the development of an epibulbar mass within months. However, there was no record of systemic involvement in that case [9].

**Table 1:** Summary of Rosai-Dorfman disease manifesting as epibulbar masses

Reference	Age/Gender	Eye	Ophthalmic findings	Extraocular involvement	Treatment	Recurrence
Zimmerman, 1988 [6]	13/M	OS	Superior epibulbar nodule (8 × 6 mm)	Minimal inguinal lymphadenopathy	Excision	No
Tan, 2002 [7]	63/M	OS	Inferior epibulbar nodule (5 × 12 mm), anterior modular scleritis, anterior and posterior uveitis	Post-auricular lymphadenopathy and vocal cord nodule	Excision + Oral & topical steroid	Yes
Albini, 2005 [8]	71/M	OD	Nasal side epibulbar nodule (15 × 15 mm)	Nil	Excision	No
	51/M	OS	Nasal side epibulbar nodule (5 mm)	Nil	Excision	No
Sarwal, 2008 [9]	53/F	OU	Superotemporal epibulbar nodule (3 × 3 mm), anterior nodular scleritis, anterior uveitis	Anterior chest wall, abdominal and pelvic masses	Excision + topical steroid	Yes
	19/F	OS	Superonasal epibulbar nodule (3.25 × 3.25 mm), nodular scleritis	Nil	Excision	No
Fernandes, 2008 [10]	19/M	OD	Temporal epibulbar nodule (12 × 11 mm), conjunctival injection	Nil	Excision	Yes
Maheshwari, 2008 [11]	17/M	OS	Temporal epibulbar nodule (3 × 5 mm)	Nil	Excision + oral steroid	No
De Oliveira, 2011 [3]	14/F	OD	Inferior epibulbar nodule (5 × 15 mm), conjunctival injection	Nil	Excision	No
Payne, 2011 [4]	20/F	OS	Inferonasal epibulbar nodule (5 × 5 mm), panuveitis, subretinal exudate	Pelvic mass	Excision + Oral & topical steroid	Yes
Shah, 2012 [12]	36/F	OS	Superior epibulbar nodule (12 × 20 mm), trace anterior uveitis	Nil	Excision + Oral & topical steroid + oral cyclosporine	Yes
Choi, 2018 [2]	35/M	OU	Epibulbar masses, retinal detachment, and choroidal effusions	Sinus, trachea, renal, subcutaneous skin lesions	Cladribine, steroids, mycophenolate mofetil, rituximab, vemurafenib	Yes
	54/F	OU	Epibulbar masses	Colon lesions, peritoneum, soft tissue of chest wall	Excision	Unknown
	41/F	OU	Epibulbar masses	Lung, aortic lymph nodes, pleura, skeletal (rib)	Steroid	Unknown
Lee, 2018 (Present case)	36/M	OS	Antecedent uveitis and anterior nodular scleritis, inferotemporal epibulbar mass	Facial skin nodule, multiple papulonodules on bilateral cheek, ear and scalp	Excision + Oral & topical steroid, methotrexate	No

Abbreviation: *F* female, *M* male, *OD* right eye, *OS* left eye, *OU* both eyes

Based on the clinical features, there are two types of RDD: one type is the classic nodal form, which is characterized by bilateral, massive, and painless cervical lymphadenopathy, and the other type is extranodal RDD [1]. Although extranodal RDD accounts for 43% of RDD cases, and the skin is involved in 10% of extranodal RDD cases [1], cutaneous RDD that only affects the skin is extremely rare [13, 14]. Whereas the histologic appearance of the cutaneous RDD is similar to that of systemic extranodal RDD, our case is a systemic RDD with multiple extranodal organ involvements.

It is theoretically possible that *Demodex* infestations might play a role in inducing RDD or aggravating the disease. *Demodex* mites parasitize healthy skin. Overgrowth of *Demodex* are found in rosacea patients, and it is speculated that the *Demodex* mites trigger the host immune response by the activation of the TLR2 pathway, leading to skin inflammation [15]. RDD coexists with inflammatory disease in 10% of cases, such as systemic lupus erythematous, erythematous, idiopathic juvenile arthritis, and autoimmune hemolytic anemia [1]. Despite the possible connection between the two disease entities, a literature review did not identify a study analyzing the relationship between *Demodex* infestations and RDD.

The standard treatment of RDD remains undetermined, although many therapeutic options, such as surgery, as well as chemotherapy, radiotherapy, oral corticosteroids, sirolimus, and immunomodulatory therapy, have been reported [1, 2, 16]. Observation of the patient rather than treatment is reasonable in many cases because up to 50% of patients with RDD have been reported to undergo spontaneous remission [1]. Nevertheless, observation alone is indicated only for patients with uncomplicated lymphadenopathy or asymptomatic cutaneous RDD and potentially for those with asymptomatic disease in other sites. Surgical excision can be curative for unifocal disease [3]; however, local recurrence may subsequently occur [4]. In our literature review, 40% (6 of 15 patients) had at least one recurrence (Table 1), and among these patients 33% (2 of 6 patients) did not receive systemic immunosuppressive therapy. The present case received various immunosuppressive regimens, including MTX as an effective maintenance therapy. The consensus recommends using low-dose methotrexate 20 mg/m^2^ per week in refractory cases [1]. Based on our experience, methotrexate cold be tapered when RDD resolves. Methotrexate could be tapered to 2.5–5 mg per week for 3–6 months as maintenance therapy. Long-term low-dose methotrexate is relatively safe, but blood counts and liver enzymes should be routinely monitored [17]. No evidence of local recurrence was noted in the present case, highlighting the importance of the administration of systemic therapy.

Cutaneous Rosai-Dorfman disease can be successfully treated with oral dapsone according to the study by Chan CC et al [18]. Chan demonstrates that numerous neutrophils and histiocytes with a positive myeloperoxidase staining are detected in specimens obtained from patients with cutaneous Rosai-Dorfman disease. As in our present case, multiple neutrophils and histiocytes were found in the pathology of the skin biopsy. Because dapsone exerts anti-inflammatory effects through inhibition of myeloperoxidase, which is present in the azurophilic granules of neutrophils, as well as in the lysosomes of monocytes, tissue-resident macrophages and histiocytes, it is one of the effective treatment options for cutaneous Rosai-Dorfman disease.

Immunosuppressive therapy could possibly worsen the *Demodex* infestations. An in vivo study reveals that *Demodex* mites rapidly colonize genetically modified mice (BALB/c-IL13/IL4) with an impaired Th2 response [19]. Thus, if the immunosuppressive therapy causes a shift from a Th2 to a Th1/Th17 immune response, it is possible the number of *Demodex* mites will increase. In a study describing dupilumab therapy as the culprit of rosacea, the author speculated that inhibition of the Th2 pathway by dupilumab may actually induce an overgrowth of *Demodex* mites and may play a role in the pathogenesis of rosacea [20].

In conclusion, we believe that scleritis and anterior uveitis should be considered as presenting signs of RDD. This subtype of extranodal RDD is characterized by a poor response to corticosteroid therapy, the formation of an epibulbar mass months later, and the absence of cervical lymphadenopathy. Because of its complexity, a multidisciplinary team is necessary for optimal patient care, and a surgical excision combined with a maintenance regimen of systemic therapy is feasible to control the disease without recurrence.

## Data Availability

All data generated or analyzed during this study are included in this published article.

## Abbreviations

MTX: methotrexate
RDD: Rosai-Dorfman disease
